# “I Am Okay With It, But I Am Not Going to Do It”: The Exogenous Factors Influencing Non-Participation in Medical Assistance in Dying

**DOI:** 10.1177/10497323211027130

**Published:** 2021-07-08

**Authors:** Janine Brown, Donna Goodridge, Lilian Thorpe, Alexander Crizzle

**Affiliations:** 1 University of Regina, Saskatoon, Saskatchewan, Canada; 2 University of Saskatchewan, Saskatoon, Saskatchewan, Canada

**Keywords:** medical assistance in dying, conscience objection, non-participation, physicians, nurse practitioners, decision-making, qualitative, interpretive description, Canada

## Abstract

Medical assistance in dying (MAID) processes are complex, shaped by legislated directives, and influenced by the discourse regarding its emergence as an end-of-life care option. Physicians and nurse practitioners (NPs) are essential in determining the patient’s eligibility and conducting MAID provisions. This research explored the exogenous factors influencing physicians’ and NPs’ non-participation in formal MAID processes. Using an interpretive description methodology, we interviewed 17 physicians and 18 NPs in Saskatchewan, Canada, who identified as non-participators in MAID. The non-participation factors were related to (a) the health care *system* they work within, (b) the *communities* where they live, (c) their current *practice* context, (d) how their participation choices were *visible* to others, (e) the *risks* of participation to themselves and others, (f) *time* factors, (g) the impact of participation on the *patient’s family*, and (h) *patient–HCP* relationship, and contextual factors. Practice considerations to support the evolving social contact of care were identified.

Medical assistance in dying (MAID) became legal in Canada in June 2016 with the royal assent of Bill C-14 (Government of Canada, 2016). Bill C-14 created the initial exemption in Canada’s Criminal Code such that physicians and nurse practitioners (NPs) can provide MAID without the charge of culpable homicide. According to the legislation, MAID is

(a) the administering by a medical practitioner or nurse practitioner of a substance to a person, at their request, that causes their death, or (b) the prescribing or providing by a medical practitioner or nurse practitioner of a substance to a person, at their request, so that they self-administer the substance and in doing so cause their own death. (p. 5)

Although legal for less than 5 years, MAID has changed end-of-life (EOL) options for patients, families, and health care providers (HCPs). At the time of its legalization, 85% of Canadians supported MAID (Ispos, 2016), and 1015 Canadians chose MAID within the first 6 months of its availability as an EOL care option (Health Canada, 2020). Despite Canadians choosing MAID at the EOL, few practitioners participate in the formal MAID processes of assessing patients for MAID eligibility and providing MAID. Previous research has examined the experiences of HCPs who participate in formal MAID processes (Beuthin, 2018; Khoshnood et al., 2018; McKee & Sellick, 2018; Shaw et al., 2018), yet there is limited data on what influences HCPs’ non-participation in the formal process of MAID. This research was guided by the question: What factors influenced physicians and NPs when deciding to not participate in the formal MAID processes of determining a patient’s eligibility for MAID and providing MAID? Identifying the factors that influence HCPs’ non-participation will foster a better understanding of the professional supports for HCPs and potential policy and practice gaps, which will therefore support patients’ care access.

## Background

### Legislative Directives of Bill C-14

Federal Bill C-14 in 2016 identified both the patient eligibility criteria and the legislated procedural imperatives to balance individual autonomy and protect the vulnerable (List 1).

**List 1.** Legislated Patient Eligibility Criteria and Procedural Imperatives.

**Patient eligibility criteria for MAID**
• Be mentally competent and at least 18 years and older
• Qualify for Canadian health services
• Provide informed consent
• Have an irremediable and grievous medical condition
• Request MAID voluntarily and without outside influence
**Procedural imperatives:**
Participating physicians and/or NPs must:
• Confirm the MAID request was in writing, signed, and dated by the patient in the presence of two independent witnesses
• Confirm the MAID request was signed and dated after a medical or nurse practitioner informed the person of an irremediable and grievous medical condition
• Independently assess the patient against the legislated legibility criteria
• Ensure the patient knew their request could be withdrawn at any time
• Allow 10 days elapsed between the written request and the provision (unless both assessors agreed that the person’s death or the loss of their capacity to provide informed consent was imminent
• Confirm consent immediately before the provision
• Ensure all measures were undertaken to ensure the patient understood the information and the patient was able to communicate their decision.

As defined within the Bill, an irremediable and grievous condition requires that (a) the disease, disability, or illness is serious and incurable, (b) the individual is in an advanced state of irreversible decline in capability, (c) the disease, disability, or illness causes intolerable and enduring physical or psychological suffering that cannot be relieved through means they find acceptable, and (d) considering all the medical circumstances, the individual’s natural death is reasonably foreseeable. Bill C-14 specified that only NPs and physicians could participate in the formal MAID processes of determining patient eligibility and providing MAID, and it additionally confirmed the freedoms of conscience and religion and called for a parliamentary review on the state of PC in Canada.

### Legislative Directives of Bill C-7

After conducting this study, Bill C-7 underwent royal assent, which altered the patient eligibility criteria and procedural safeguards, included provisions for final consent waivers and advanced consent, outlined additional reporting/monitoring requirements, and called for additional parliamentary reviews (Government of Canada, 2021). Specific changes include (a) removal of the requirement for a reasonably foreseeable death required, (b) specification of different procedural safeguards for track one requests, when death is deemed reasonably foreseeable, and track two requests, when death is not deemed to be reasonably foreseeable, (c) inclusion of mental illness as the sole illness, disease or disability for the purposes of eligibility on the second anniversary of the Royal Assent (March 16, 2023), (d) provisions for a final consent waiver for those patients who have been assessed and approved for MAID, have set a date for MAID and are concerned about the loss of capacity before that time), and (e) provisions for an advanced consent for those patients who do not die within a specified period after self-administration of MAID medications, the HCP could proceed with intravenous MAID.

### MAID Programming in Canada

Bill C-14 and the amendment of the Criminal Code of Canada was a change in federal law. However, Canadian provinces and territories are responsible for health care delivery, and as such, provincial/territorial and regional health care MAID program delivery varies across Canada (Pesut, Thorne, Schiller, et al., 2020; Wiebe et al., 2020). Variations may be related to differences in population values, interests and resources, provincial/territorial contexts and indicators, and diversity in existing health care delivery structures (Silvius et al., 2019). Health care systems are in various stages of developing accessible, high-quality MAID programs that are patient-and-family centered and sustainable within various care models. Some have incorporated MAID into existing HCP workloads, some have devised patient care pathways, some have implemented standard access processes and medication protocols, and some have centralized case coordinators to support patients, families, and providers (Silvius et al., 2019; Wiebe et al., 2020).

### The Saskatchewan MAID Program

This research was conducted in the province of Saskatchewan, Canada, where 38% of the approximate 1,170,000 population was located in rural and remote areas (Statistics Canada, 2016). At the time of data collection this population was served by 267 NPs and over 2,600 provincially licensed physicians (Saskatchewan Ministry of Health, 2019; Saskatchewan Registered Nurses Association, 2018), although interview data might have been based on experiences prior to this. Since legalization, there have been 250 MAID provisions in Saskatchewan (Health Canada, 2020). At the time of this study, health care delivery in Saskatchewan was the responsibility of a single, publicly-funded health authority. The provincial MAID program, which came into effect in November 2018 (Bridges, 2019), had salaried employees and an NP in each of the two largest cities. On a case-by-case basis, these NPs and other NPs and physicians conducted MAID eligibility assessments and MAID provisions across the province. Although prior to the development of the provincial MAID program referrals generally came directly to MAID assessors from another HCP, by the time this study was conducted, the main referral pathway to the provincial MAID program for HCPs was through the provincial Healthline. This meant that patients and family members were able to access the provincial MAID program directly without a physician or NP referral. MAID assessors have been able to assess patients across the province either in person or via the use of technology. MAID provisions have occurred in multiple settings which were agreeable to the patient, provider, and, as necessary, the institution.

According to the provincial MAID program, between November 2018 and February 2020, 35 (or 0.012%) NPs and physicians have participated in the formal MAID processes of assessment and/or provision, with approximately half participating in fewer than five occurrences (M. Fisher, personal communication, February 27, 2020). Conscientious objection (CO) is embedded in provincial professional regulatory association statements (College of Physicians and Surgeons of Saskatchewan, 2017; Saskatchewan Registered Nurses Association, 2016a, 2016b).

### Palliative and EOL Care in Canada

Palliative care (PC) is a holistic care approach that (a) seeks to improve the quality of life for patients and families with life-threatening illnesses, (b) intends “neither to hasten or postpone death,” and (c) should be “integrated with and complement prevention, early diagnosis, and treatment” of health challenges (World Health Organization, 2018, p. 5). In Canada, the term “hospice palliative care” recognizes the convergence of PC and hospice care convergence because of principles and practice norms (Canadian Hospice Palliative Care Association [CHPCA], 2013). Sercu et al. (2018) identified a framework of four PC phases, which included the advanced illness phase, the EOL phase, the terminal phase, and the dying phase, and Funk et al. (2017) noted PC providers often “struggled to find the time and space to deal with grief and [are] faced normative constraints on grief expressions at work” (p. 2211).

By the stated definition of PC above, PC philosophically diverges from that of MAID, which actively hastens death to decrease suffering. Despite this philosophical divergence, Wales et al. (2018) reported successful integration of MAID into home-based PC, and Dierickx et al. (2018) found that assisted dying, and PC were not “contradictory practices” (p. 114). However, the co-existence of MAID and PC within EOL care in Canada is viewed differently among the CHPCA, the Canadian Society of Palliative Care Physicians (CSPCP), and the Canadian Association of MAID Assessors and Providers (CAMAP). The CHPCA and CSPCP (2019) believe that MAID is not part of hospice PC practice as they are fundamentally different, whereas CAMAP (2020) encourages the integration of PC and MAID. Understanding these differences in the fundamental beliefs related to EOL care is essential because HCPs’ response to MAID inquiries is influenced by their conceptualization of MAID relative to other EOL care options (Seller et al., 2019).

### Freedom of Religion and Conscience and Moral Convictions

#### Freedom of religion

The preamble of Bill C-14 upholds section 2 of the federal Canadian Charter of Rights and Freedoms (Government of Canada, 2016), which guarantees freedom of conscience and religion. Freedom of religion is defined by the Supreme Court of Canada (1996) as:

The right to entertain such religious beliefs as a person chooses, the right to declare religious beliefs openly and without fear of hindrance or reprisal, and the right to manifest religious belief by worship and practice or by teaching and dissemination. (p. 868)

Medicine, religion, and spirituality share an extended narrative, including priests’ historical role as healers, hospitals founded by religious organizations, and the values of compassionate service (Sajja & Puchalski, 2018). Practicing in alignment with religious or spiritual views is an essential component of moral integrity (Wicclair, 2011). A review of Christianity, Islam, Buddhism, Hinduism, and Judaism beliefs relative to EOL practices (including assisted dying) found significant deficits in the available knowledge base, identified dramatic variations in subpopulations studied (Chakraborty et al., 2017). It also noted the influence of national cultural practices and laws on religious perspectives and practices.

#### Freedom of conscience

While freedom of religion has been given “extensive legal attention,” freedom of conscience is often forgotten (Bird, 2017, para. 4). The values that shape conscience (i.e., fair or unfair, just or unjust) are influenced by an individual’s cultural, economic, and political environments (Vithoulkas & Muresanu, 2014). Conscience is “an internal moral decision-making process that holds someone accountable to their moral judgment and for their actions” (Lamb et al., 2019, p. 1338), and freedom of conscience allows individuals to “manifest their moral commitments” (Bird, 2017, para. 5). According to Wicclair (2011), moral integrity has intrinsic value as it is an essential component of a meaningful life, and a loss of moral integrity can result in a loss of self-respect, feelings of shame, remorse, or guilt, and a decline in moral character. As such, both freedoms of conscience and religion are critical to HCPs and health care delivery.

#### Moral convictions

HCPs also work within their moral convictions and the cooperative behaviors that underpin universal moral rules. Moral convictions, or “attitudes that people perceive as grounded in a fundamental distinction between right and wrong,” (Skitka et al., 2021, p. 347) guide HCPs in determining their participation in care. In addition, HCPs also are influenced by the cooperative behaviors (i.e., helping your family and group, fairing dividing resources) that underpin universal moral rules (Curry et al., 2019). Harmonizing these considerations may result in HCPs choosing not to participate in the care requested by the patient, or in other words, choosing not to participate in legally available care.

### Conscientious Objection and Non-Participation

Professional associations and regulatory bodies include CO or respect for freedom of conscience statements in their MAID practice policies and frameworks (Canadian Medical Association, 2016; Canadian Nurses Association, 2017). However, Wicclair (2011) explained that not all refusals to participate are grounded in HCPs’ core moral beliefs or conscience and that reasons for refusing can include self-interest and professional integrity. HCPs’ non-participation in ethically complex, legally available care was influenced by their characteristics, personal beliefs, and professional ethos, as well as emotional labor, system, and clinical practice considerations (Brown, Goodridge, Thorpe, Hodson and Chipanshi, 2021). Thus, it is crucial to fully explore the underlying factors contributing to conscience claims so that conscience claims are not used to avoid care that is prejudicial, time-consuming, emotional, or discriminatory (Brindley, 2017; Lachman, 2014). Specific to MAID, the emotional burden of care participation, the concern regarding psychological repercussions, as well as moral and religious grounds, were the most often expressed reasons that physicians conscientiously objected (Bouthillier & Opatrny, 2019). In addition, we previously reported that the endogenous factors that influenced non-participation in formal MAID processes included HCPs (a) previous personal and professional experiences, (b) level of comfort with death, (c) faith or spiritual beliefs, (d) preferred EOL care approaches, (e) self-accountability, (f) the consideration of emotional labor, (g) concern regarding the future emotional impact, and (h) conceptualization of professional duty (Brown, Goodridge, Thorpe and Crizzle, 2021). Collectively this research shows that conscience, religion, and other non-conscience-based factors influence HCPs’ non-participation in formal MAID processes.

Health care institutions associated with religious groups have some policy autonomy. As such, some theorize that CO could extend to health care institutions (Christie et al., 2016). However, within the Canadian publicly funded health care system, this has been challenged (Weichel, 2020). Bill C-14 does not directly state that MAID must be available in all health care facilities; however, it was recommended that health care facilities allow MAID assessments or provisions or facilitate patients’ safe transfer to an alternative health care facility (Gibson & Taylor, 2015).

## Theoretical Frameworks

We considered HCPs’ non-participation in MAID processes within the context of Social Contract Theory and Ruggiero’s (2012) model of moral decision making. A social contract is an agreement between groups in society for mutual benefit (Waugh, 1993). Health care professions use social contracts to establish their identity and outline their relationships with society (Rochelle, 1983). Social contracts between society and HPCs are fluid and shift with changing professional standards, laws, patients’ needs, and advancing patient expectations as society diversifies (Waugh, 1993). Thus, with the royal assent of Bill C-14, the social contract of care between patients and HCPs has evolved.

The Ruggiero (2012) model can be used to explore moral decision-making within individuals’ obligations, moral ideals, and consequences of their decision. He identified that individuals seek actions that minimize negative consequences and align with their ideals and obligations. Obligations are affected by relationships (including friendship, colleagueship, or business relationships) and formal and professional responsibilities. Moral ideals are the ethical values and religious values that assist in achieving respect for persons. The consequences of the decision encompass the actual, possible, or probable, beneficial, or harmful outcomes. These consequences could be physical or emotional, immediately apparent or apparent over time, intended or unintended, or readily apparent, subtle, complex, or specific.

## Method

This research was grounded in a constructivist/interpretivist paradigm, and we acknowledge that our interpretations are specific to our research team, setting, time, and the participants. We acknowledge there are socially constructed, sometimes conflicting realities (Ponterotto, 2005) and that these realities may change as individuals change (Guba & Lincoln, 1994). We used interpretive description (Thorne, 2016), which addresses the research objective by capturing and interpreting the participants’ perceptions, seeking patterns, and generating themes to create applied knowledge that informs clinical care. Janine Brown led the research with the support of the co-authors and a doctoral committee. The authors frequently met to discuss underlying and emerging views and perceptions that supported the team’s reflexive processes during the research process.

### Sampling Strategy

Provincially licensed physicians and NPs who self-identified as (a) being uncertain of their response to a patient request for MAID assessment or provision, (b) being reluctant to engage in MAID related processes, or (c) declining participation in any aspect of MAID were invited to participate in this research. We excluded HCPs who practice exclusively with patients under the age of 18. We initially planned to interview 40 participants representing variation in geographic location, profession, practice patterns, and participant demographics. We employed multiple strategies for participant recruitment. We asked the physician and NP regulatory bodies and professional associations, the medicine and nursing university faculties, the division of northern medical care, the provincial health authority, and the cancer agency to distribute ethics-approved invitation letters, posters and, social media scripts. In addition, consenting individuals and doctoral committee members were asked to forward the research information through their networks. Potential participants contacted Janine Brown (the interviewer) via email. Janine Brown confirmed the participant’s eligibility and sent the potential participants the information and consent form. If the participants chose to proceed, a mutually agreeable time and interview modality were determined. Janine Brown obtained verbal consent during the interview and confirmed consent on a written consent form. Participants confirmed consent on the online contextual information questionnaire.

### Data Production

This research included participant contextual data, participant interview data, and the field notes and reflective content produced by Janine Brown. Contextual data were collected via an online questionnaire, which was completed before or during the interview. This data was collected to gauge the sample’s representation during data collection and frame the participants’ personal and practice contexts within the data. Interview data were collected using a semi-structured interview guide and vignettes informed by our theoretical frameworks (Supplemental File 1). Vignettes were chosen to support the exposition of participants’ attitudes, perceptions, beliefs (Hughes & Huby, 2002), and decision-making processes (Evans et al., 2015). The use of vignettes was essential to our data collection, as we were aware that not all participants might have had experience in MAID or patient MAID inquiries. The vignettes encompassed multiple aspects of MAID and were developed through the team’s clinical and practice experiences and reviewed by two NPs and two physicians to support validity before use. We read the vignettes to the participant, allowed the participant to respond, and followed up with exploratory or clarifying questions as required. After four interviews, we reviewed the data to ensure the exposition of the research’s objective. No vignette adjustments were made. After each interview, Janine Brown produced field notes, with notations on the data collection event itself, and reflections on emerging perspectives, striking and illuminating content, and emerging questions to bring forward to the next interview. This supported researcher reflexivity and informed future interviews, data analysis, and interpretation.

### Ethics and Operational Approval

We received research ethics (REB#902) and provincial health authority operational approval (OA-UofX-902) for this research. We made it clear that the doctoral committee would access the data within the ethics approval, and we identified procedures for sharing the aggregate data with the participants. We indicated that the research team members might have pre-existing relationships with potential participants. However, we would not exclude them, as our health care community is relatively small, and these relationships are professional. Finally, recognizing the topic’s potentially sensitive nature, we provided the participants with information on how to access professional support through their professional association or employer.

### Data Interpretation

We used NVivo12 to organize the transcripts, contextual data, field notes, and reflective content. With the support of the co-authors, Janine Brown concurrently collected and analyzed the data. Using a process of inductive coding as outlined by Boeiji (2002), coding was conducted within a single interview, followed by code comparison between interviews and, finally, across the entire data set. Janine Brown developed the initial patterns of meaning and shared them with the participants with an invitation to provide any additional information, insights, comments, or reflections. Subsequently, these initial patterns underwent combining, refining, and eventual interpretation and theming (Braun & Clarke, 2019). Documents outlining the resultant themes, definitions, and supporting participant quotations were cross-checked by the co-authors and presented to the doctoral committee as part of an expert panel analysis check (Thorne, 2016).

### Quality and Credibility

Research quality and credibility were given high priority throughout the research. We aligned our methods with our methodology and accounted for our positionality and reflexivity. We included multiple data sources, vetted and trialed the vignettes, and used a single transcriptionist and primary coder. Donna Goodridge and Lilian Thorpe cross-checked the codes, and documents were utilized to account for the results. Finally, the results were shared with the participants, and feedback was obtained from an expert panel review.

## Results

We determined that we had adequate data to fulfill our research objective and found a broad representation of contextual data after 35 interviews (see Supplemental File 2 for a complete demographic and contextual report). In response to the vignettes, all HCPs stated they would refer the patient to the MAID program through the provincial referral pathway or direct the patient to speak with an alternative HCP. However, notably, few HCPs participating in our study could articulate the MAID program referral pathways. Fourteen HCPs identified that referring to the MAID program or directing the patient to speak with an alternative HCP was their participation threshold. In contrast, the remaining HCPs anticipated alternative degrees of participation (i.e., they anticipated they could discuss MAID as an EOL care option or could provide emotional support on the day of death for the patient and family) in the clinical care vignette. None of the HCPs stated that they would participate in the provision of MAID.

### Exogenous Influencing Non-Participation

A spectrum of factors influenced HCPs’ non-participation in formal MAID processes. While recognizing that decision-making is generally thought to be an intrinsic process, through the data analysis, some of the factors identified by the participants were related to external conditions or circumstances. These were conceptualized as exogenous factors.

**Figure 1.**
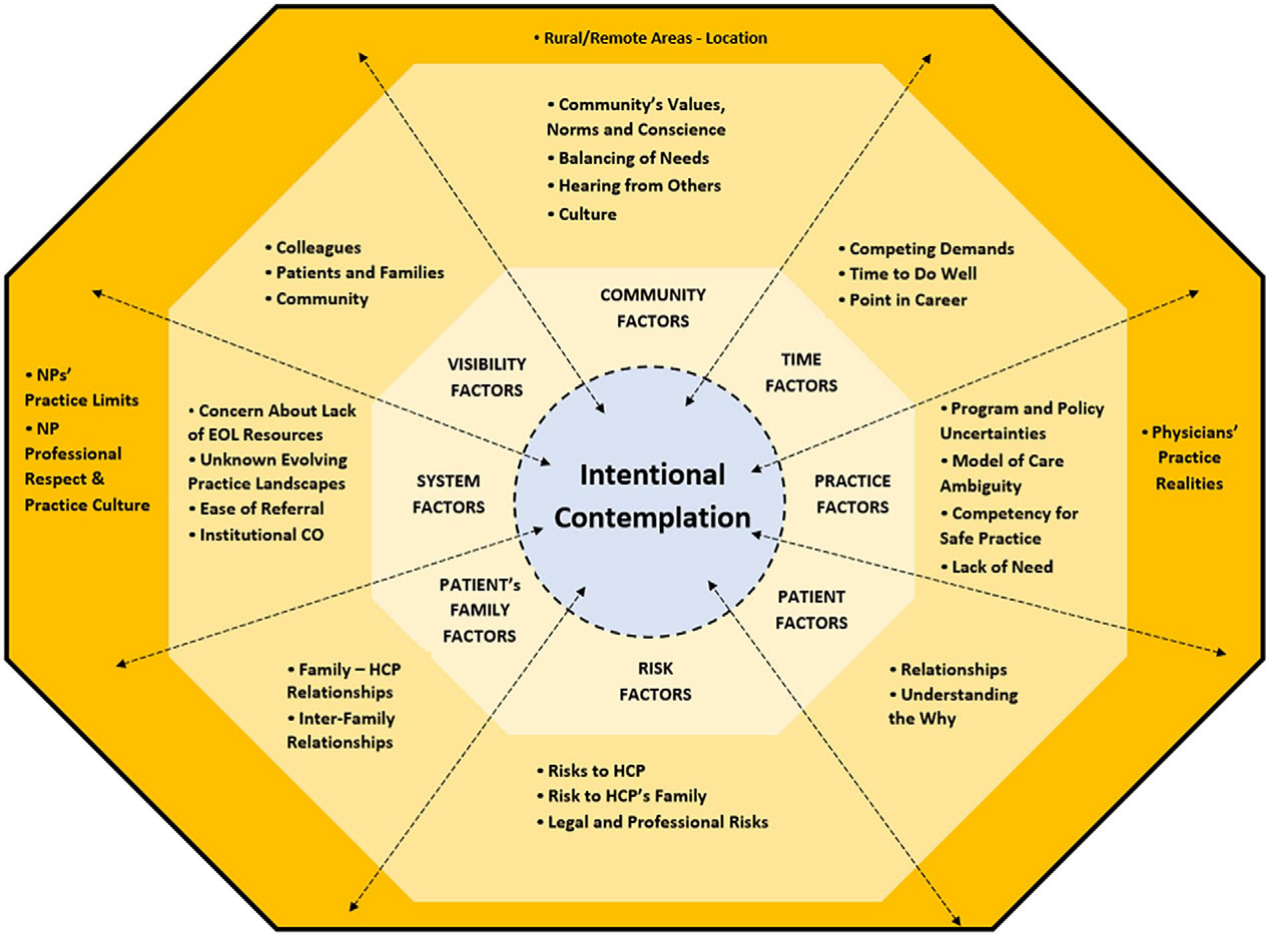
Exogenous factors influencing non-participation in formal MAID processes. *Note.* MAID = medical assistance in dying; NP = nurse practitioner; EOL = end-of-life; CO = conscientious objection; HCP = health care provider.

We found eight exogenous factors that influenced HCPs’ non-participation in formal MAID processes. These factors were identified as consistent themes across the data and were related to (a) the health care *system* they work within, (b) the *communities* where they live, (c) their current *practice* context, (d) how their participation choices were *visible* to others, (e) the *risks* of participation to themselves and their family, (f) *time* factors, (g) the impact of participation on the *patient’s family*, and (h) *patient–HCP* relationship, and contextual factors. HCPs identified multiple decision-making considerations within each factor. Some of the decision-making considerations were nuanced to specific demographics, including the HCP’s practice location and the HCP’s professional group (Figure 1).

#### The health care system they work within

HCPs’ non-participation in formal MAID processes was influenced by the health care system HCPs work within. Specifically, HCPs considered (a) their concern about a lack of EOL care resources, (b) an uncertain and evolving practice landscape, (c) the ease of referral, and (d) institutional CO. In addition, NPs considered their employer-imposed practice limits, professional respect, and practice culture.

Some HCPs’ identified that their non-participation in formal MAID processes was influenced by their concern regarding gaps in the current provision of EOL and chronic care. These HCPs explained that before they could consider participation in formal MAID processes, these system gaps required remediation. Specifically, these HCPs raised concerns about the limited access to palliative and chronic care support in outpatient, inpatient, and respite settings:

I never want to suggest that conversations [about MAID] should never be on the table, so I am reluctant to make that argument. At the very least, could we be doing an impeccable job of chronic care support and disease management and palliative care first? Doing all of those things impeccably well, for every Canadian, and then if we still need it, well, maybe we could talk.

Other HCPs identified that their non-participation was influenced by the “newness” of MAID as an EOL care option and the associated evolving and uncertain practice landscape. This resulted in a reluctance to participate until more Canadian experience with models of practice or evidence to support this evidence-based clinical care area became available. In addition, for some HCPs simple referral processes and personal connections with existing MAID assessors and providers were considered “easy” referrals that facilitated HCPs’ disengagement from participation:

So, it is easy for me to say to patients, “We have to refer you [for formal MAID processes] through the centralized process to the next regional center.” It is easy for me to say that. So, it gives me a bit of an out.

Some HCPs were frustrated that their non-participation was determined by institutional CO, which occurred when faith-influenced institutional policy directives prohibited MAID participation or limited their practice (“We have buildings where [MAID] cannot be practiced . . . personal beliefs should not restrict or be the gate-keeper to patient care or what practitioners want to provide”). However, other HCPs identified institutional CO meant they did not need to discuss their motivations or belief systems with others and could avoid participation. For these non-participating HCPs, faith-influenced institutional policy directives provided a source of comfort.

Specific to some NPs, employer practice limits that affected NPs’ ability to participate in formal MAID processes resulted in their non-participation. The practice limits that affected NPs’ ability to practice to their full-scope and included (a) absence of billing codes for remuneration, (b) agency job descriptions that limited care duties or excluded MAID participation, (c) an inability to roster patients in their practice resulting in episodic or singular care encounters, and (d) an inability to admit patients to hospitals resulting in patients with life-limiting illnesses being transferred to physicians. Some NPs’ non-participation was also influenced by their frustration regarding the culture of their practice. Specifically, some NPs described frustration at being overlooked during the early stages of MAID delivery as assessors and providers. They felt their participation only appeared to be considered when the availability of physicians was scarce, and there was a perceived lack of professional respect from physician colleagues and health system administrators.

#### The communities where they live

HCPs’ non-participation in formal MAID processes was influenced by the communities where they lived. Specifically, HCPs considered (a) the community’s values, norms, and conscience, (b) how they needed to balance the needs of the entire community, (c) what they heard from others within their community about MAID, and (d) the integration of culturally safe practices. Some HCPs stated that their non-participation in formal MAID processes was influenced by their perception of the community’s conscience and used community cues to gauge participation’s appropriateness. These community cues included (a) a lack of openness in other EOL conversations (i.e., “we don’t even talk about DNRs here!”), (b) a lack of sexual health programs and services, which resulted in HCPs’ hesitation to bring forward ethically complex conversations, (c) the communities’ perceived dominant religious beliefs, (d) the relationship between the HCPs and the community, and (e) the community’s history with suicide and suicide prevention initiatives resulting in sensitivity or potential mixed messages in MAID conversations.

Some HCPs’ non-participation was also influenced by the potentially adverse impact of competing demands. Specifically, HCPs considered how participating in one individual’s care (i.e., participating in formal MAID processes) would affect their ability to meet the greater community’s care needs. These HCPs were ethically concerned about the prospect of declining, decreasing, or canceling service in an already limited setting, which would be required to facilitate participation in formal MAID processes:

NPs work in small centers that get service two days a week. So, to take a half a day out of what is already limited service is very difficult and somewhat angst producing for the NPs who feel ethically and morally responsible for the lack of services in those areas.

In addition, some HCPs’ non-participation in formal MAID processes was influenced by the adverse experiences of others in their professional or home community related to MAID participation (“I have sort of talked about it with one of the NPs that has [participated in formal MAID processes], she has struggled, and it is not something you can take back”).

Finally, HCPs identified their non-participation in MAID processes as influenced by the complexities of working within culturally diverse contexts. These HCPs were hesitant to participate in formal MAID processes as they were unsure *if* or *how* the community’s culture influenced the perception of MAID and if participation in MAID would alter the community’s trust in them. Some HCPs noted that using interpreters significantly complicated EOL conversations and discussed the anticipated exponential difficulties of using interpreters in formal MAID processes. These HCPs related situations when interpreters refused to translate or when the interpreters filtered the HCPs’ discussions. In addition, they expressed concern regarding patient confidentiality, as translators were often family members or extended family members. Finally, in rural and remote areas, HCPs anticipated that if they did not support, facilitate, or participate in formal MAID processes, there would be “undue burdens” on patients and families, who would need to travel to another center and would experience increased costs. These HCPs also expressed concern that these considerations would add extra pressure to participate, which they factored into their participation perspectives:

Within the [Indigenous] population that I work with, I want to make sure that I am not overstepping my boundaries of trust by being [involved with MAID], or that it would be seen as disrespectful. I do not ever want it to cause distress to the patient.

#### Their current practice context

HCPs’ non-participation in formal MAID processes was influenced by their current practice context. Specifically, HCPs considered the influence of (a) an ambiguous model of care, (b) program and policy uncertainties, (c) their competence to provide care, and (d) a perceived lack of need within their practice.

Some HCPs’ non-participation was influenced by their uncertainty about the optimal regional MAID model of care (“I just do not know where putting that kind of specialized care and knowledge would go!”). Many questioned whether MAID was a component of family practice, an extension of existing EOL care programming, or a specialty practice area. The ambiguity of not knowing *if or how* MAID fit within their practice influenced their prioritization of MAID continuing education and their overall participation perspectives. Other HCPs’ non-participation in formal MAID processes was influenced by their lack of operational program and policy knowledge:

“How do you pronounce death? What do you put on the certificate [after MAID]?” Regardless of what we think about MAID, you know, there are very real practical issues that you have to resolve regardless of your personal feelings [before considering participation].

Some HCPs’ non-participation in formal MAID processes was influenced by their lack of skills, abilities, and competencies to participate in formal MAID processes. These HCPs expressed uncertainty about (a) applying the eligibility criteria to their patients, (b) utilizing medication protocols, (c) navigating sensitive or challenging conversations, (d) understanding what competency in MAID encompassed, and (e) maintaining competency if infrequently participating. Other HCPs’ non-participation was influenced by their perceived practice strengths and their belief that it was unlikely that patients would approach them in MAID discussions (“I have not had a lot of, even motivation, I suppose, to look into it just based on where I practice”). Some physicians’ non-participation in the formal MAID process was influenced by their practice constraints, specifically the financial feasibility of participation in formal process relative to their operational overhead costs and the cost of malpractice insurance:

I know a few colleagues of mine said financially they cannot offer [MAID]. You can be out doing [MAID] for four hours, make $100, lose a half a day in clinic, and pay six, seven grand in overhead clinic costs. You are not making your ends meet doing that. Family practice right now is stretched financially.

#### How participation was visible to others

How colleagues and clinic staff would view their participation or non-participation in formal MAID processes influenced HCPs. Some HCPs feared collegial disapproval if they did not participate, and some feared their non-participation would be viewed as shirking their professional duties or viewed as acting counter to patient autonomy. Other HCPs believed that if they participated in formal MAID processes, they would lose the clinic staff’s respect or were concerned about how colleagues of the same faith would view them. In addition, some HCPs expressed “surprise” when colleagues participated in MAID and that this changed their perceptions of their colleagues. They wondered how their colleagues could participate and discussed how they viewed their colleagues’ practice approaches differently:

I have also talked to physicians who get angry at the talk about conscientious objection. They feel that, you know, physicians are not doing their job, that they are shirking their responsibility.

As patients and families are not obligated to maintain HCPs’ privacy regarding their participation, HCPs considered how participating in formal MAID processes could influence how members of the public viewed them (“I worry about how patients would feel about their practitioner being involved in this process”). Specifically, some HCPs were concerned that being known as participating in MAID would harm the relationship with patients and families who object to MAID, that participation would be interpreted as “giving up” on patients, or that participation would complicate mental health and suicide prevention conversations. Finally, some HCPs’ were concerned that the greater community or their faith community would view their participation unfavorably, which would affect the relationships with others therein:

I just could see some people who might have suicidal ideations saying to us, “You are a hypocrite. How can you try to tell me [suicide] is wrong or that I should not do this when you are doing it? You did it to my granny.”

#### The risks of participation

HCPs’ non-participation in formal MAID processes was influenced by their concern regarding the risk that participation might pose to themselves, the professional risk of litigation or professional discipline, and the risk to their families. First, HCPs considered the risk of personal physical harm or violence from extended family members or the risk that their professional lives could be made “difficult” by colleagues if they participated in formal MAID processes:

You know, when I have had to discuss death with a whole bunch of family members, I have seen people’s responses go from very calm to very violent within a split second of me saying they died. It has never been towards me, but if I am the one who is pushing the injection, then it might be towards me.

Other HCPs were concerned about the risk of litigation or professional discipline if family members or other HCPs disagreed with the patient’s choice or the HCPs’ eligibility assessments (“I am okay with it [MAID], but I am not going to do it and risk my license!”).

And finally, some HCPs’ identified that their non-participation was influenced by a concern for their family’s safety:

I am more worried about my family than myself. We have already had some backlash in the community where lawyers were involved. I had to take my kids out of town, and maybe this is worse case catastrophizing, but it happened. We have some very religious people, and we have people with lots of guns, and I would not take that risk with my kids.

#### Time factors

HCPs’ non-participation in formal MAID processes was influenced by time. In particular, HCPs considered their multiple time demands against the time required for quality MAID care and the time they were at with their career. Some explained that competing demands in time-limited appointments resulted in insufficient time for EOL conversations and participation in formal MAID processes (“I do not have the time to do it”). In addition, HCPs identified that participation in formal MAID processes should not be rushed, and the lack of time to participate in quality care limited their participation (“If I cannot do it well, then I do not want to take it on”). Some HCPs’ explained that their non-participation in MAID was relative to the time of their career. Some identified as not wanting to take on new “challenges” at the end of their careers, whereas others stated they would re-evaluate their future participation. Finally, some HCPs’ noted that time constraints also prohibited pursuing continuing education in MAID:

The only thing [keeping me from participating] is my age and being close to retirement. I am 59 and might be pulling this [retirement] plug at the end of the year. So, to me, that is why I thought, well, I am not going to bother.

#### The impact of participation on the patient’s family

HCPs’ non-participation in formal MAID processes was influenced by their concern that their participation would affect the family member–provider relationships as they also provided primary care to extended family members. For other HCPs, inter-family conflict and a lack of supports for family members before, during, and after MAID provision influenced their non-participation. Finally, some HCPs were concerned that their MAID participation would have a lasting impact on internal family relationships and dynamics:

That whole family dynamic piece, like, “Mom is really suffering. We don’t want her to suffer.” Or, “I don’t want Mom to die yet. It is not time for Mom to die yet. Mom should not die yet.” Those pieces . . . there will be so much dealing with the family through the grief process, the blame game, the what-if game.

#### Patient–HCP relationship and contexts

HCPs’ non-participation in formal MAID processes was influenced by their relationships with their patients and the need to understand the context of the patient-specific journey. For some HCPs, a long-standing relationship with the patient would render participation “uncomfortable” and unlikely. Conversely, other HCPs identified that lack of sustained, deep relationship with the patient or family would influence their non-participation (“I do not think I should be doing it in my practice should be doing it because I do not have those relationships with people”). However, others identified that a long-standing relationship would facilitate open conversations regarding the reasons precipitating the HCP’s need to disengage from formal MAID processes. HCPs expressed a need to have a comprehensive understanding of the patient-family journey, including the clinical history and decision-making processes that culminated in their MAID choice. These factors were considered important to the HCP’s perspectives on their non-participation:

It is like no different than if I am asking them why they are not taking their diabetes medications. I want to know, “okay, so I noticed that you are choosing not to take all of these medications. What is going on? Can you help me understand?” In the [MAID] regard, it would be, “Yes, I am happy that you brought up the topic, and I am happy to put you in contact with people who can provide you with this information. But, I also want to clarify, you know, your thoughts behind that choice as opposed to other end-of-life care options.”

## Discussion

We considered HCPs’ non-participation in formal MAID processes within the context of Social Contract Theory and Ruggiero’s (2012) model of moral decision making. In doing so, we themed eight exogenous factors that influenced physicians and NPs when deciding not to participate in the formal MAID processes of determining a patient’s eligibility for MAID and providing MAID. The identified that exogenous factors that influence HCPs’ non-participation in formal MAID processes were related to (a) the health care *system* they work within, (b) the *communities* where they live, (c) their current *practice* context, (d) how their participation choices were *visible* to others, (e) the *risks* of participation to themselves and their family, (f) *time* factors, (g) the impact of participation on the *patient’s family*, and (h) *patient–HCP* relationship and contextual factors.

As Seller et al. (2019) noted, HCPs’ responses to MAID inquiries are influenced by their conceptualization of MAID relative to other EOL care options. This was evident in our findings in HCPs’ concern regarding a lack of adequate EOL care resources. In addition, Wicclair (2011) explained, “matters of conscience involve a particularly important subset of an agent’s ethical or religious beliefs—*core* [Wicclair’s emphasis] moral beliefs” (p. 4). The ability of HCPs to work within conscience is recognized in the various Canadian and provincial practice statements supporting HCPs ability to enact a CO (Canadian Medical Association, 2016; Canadian Nurses Association, 2017; College of Physicians and Surgeons of Saskatchewan, 2018; Saskatchewan Registered Nurses Association, 2016b). However, as evident in our findings and the results of Bouthillier and Opatrny (2019), non-participation in MAID may not always be rooted within Wicclair’s definition of conscience or the HCPs’ religious views. As such, professional regulators must clarify HCPs duty of care in the event of non-participation for reasons other than conscience. In single provider and rural/remote practice settings, this clarification is acutely required to support patients’ and families’ access to all care options at the EOL. Bill C-14 confirmed HCPs’ freedom of conscience and religion, but interpreting what that means for institutional CO remains uncertain. Our results identified that institutional CO is one of the factors HCPs consider in their non-participation in MAID. Shaw et al. (2018) also noted that refusals of faith-based health care institutions support MAID processes was a structural and emotional challenge. However, Shadd and Shadd (2019) explored there may be significant considerations why not all health care centers participate in MAID.

We noted in our results that the newness of MAID, the evolving practice landscapes, and the resultant uncertainty in programs, policies, and models of care influenced non-participation. Bill C-7 passed in March of 2021, and this legally driven care area is unfolding and unique. It is reasonable to consider that these legislative, policy, and practice changes will continue to influence HCPs’ non-participation until there is some consistency in practice. That being said, Bellens et al. (2020) reported that 15 years after euthanasia was legalized in Belgium, nurses still characterize their involvement in the euthanasia process as “intense and not unambiguous” (p. 495). Finally, Stewart et al. (2021) conducted their study while the Canadian Parliament was still considering the eligibility changes of eventual Bill C-7. They found that participating HCPs believed alternations to the eligibility criteria would likely result in additional patient family conflict and clinical load, and 20% of participating HCPs in their study identified they considered stopping MAID work.

There is emerging data on the motivations of Canadian HCPs who are participating in formal MAID processes. Oliphant and Frolic (2020) explored the factors that contributed to conscientious participation in MAID. They explained that the motivations for participation could be categorized into (a) personal values and identity, (b) professional values and identity, (c) experience with death and dying, and (d) influencing all the social contexts where MAID occurs. Pesut, Thorne, Storch, et al. (2020) noted that willingness to participate in MAID was influenced by nurses’ (a) family and community influences, (b) professional experiences, and (c) proximity to the act of MAID. Our results (related to both the endogenous and exogenous factors) align with these studies as individuals are choosing their degree of MAID participation based on organizational factors, family and community factors, previous personal and professional experiences, and their values individuals and professionals.

### Integration of Theoretical Frameworks and Intentional Contemplation

We conceptualize intentional contemplation as the manner in which HPCs frame the factors influencing their non-participation relative to the consequences of their participation, their moral ideals, and their obligations. It further represents the process of considering the multiple, complex, and often inter-related exogenous factors that influenced HCP’s non-participation in formal MAID processes in an evolving social contract relative to their current clinical practice context. The process of intentional contemplation reflects the profound and purposeful HCP deliberation of how their current professional practice does not integrate with participation in formal MAID processes.

MAID has shifted the social contract of EOL care, and these factors and decision-making considerations are under intentional contemplation by HCPs. For the participants in our research, this culminated in non-participation in formal MAID processes. However, all participants would facilitate the social contract of care by referring to the MAID program (if they knew the MAID program referral pathway) or an alternative HCP (if they did not know the pathway). In this sense, the social contract of care is fulfilled. However, not all HCPs in our research study could identify the referral pathways. As such, referral pathways must be adequately communicated to all health care team members, patients, and families, *and* be attentive to HCPs’ moral space to truly facilitate the social contract of care (Brown, Goodridge, Thorpe and Crizzle, 2021).

Ruggiero (2012) explained that individuals choose actions that support their obligations, support their ideals, and have favorable consequences. HCPs in this research study intentionally contemplated their professional *obligations* relative to (a) their ongoing care duties to the patient’s family, (b) institutional CO, (c) their role in the regional model of MAID care with a continually evolving practice and legal landscape, (d) their lack of skills, abilities, and competence to participate in formal MAID processes, (e) the ease and ability to refer, (f) current time and place of their career, (g) their practice limits and realities, (h) their belief that it was unlikely a patient would approach them for MAID discussions in their practice, and, (i) their concerns regarding the scarcity of non-MAID EOL care resources. In addition to their professional obligations, HCPs also intentionally contemplated their obligations to their families and communities. The intentional contemplation of *moral ideals*, or concepts that assist in achieving respect for persons (Ruggiero, 2012), was evident as HCPs intentionally contemplated (a) a lack of time to participate in what they would deem quality EOL care, (b) the need to contemplate and integrate what they hear from the experience of others, (c) the need to practice within the conscience of the greater community, (d) the cultural nuances in EOL care, (e) the need to understand the patient’s care history and decision-making, (f) the importance of the patient–HCP relationship and, for NPs (g) the need to achieve professional respect within the current practice culture. HCPs intentionally contemplated an extensive array of participation *consequences*, including (a) reduced available time to care for the patients in their practice to have adequate time to participate in MAID, (b) the consequences of professional association discipline, (c) litigation, (d) harm to themselves or their families, (e) being known or being visible as a care participator by their colleagues, other patients, and the greater community, (f) the impact on the patient’s family unit after MAID provision, and (g) undue burdens on patients and families in rural areas.

### Endogenous and Exogenous Factors

The exogenous factors should be considered in tandem with the previously reported endogenous non-participation factors (Brown, Goodridge, Thorpe and Crizzle, 2021) to support a comprehensive understanding of the factors influencing non-participation in the formal MAID process of assessing patients for and providing MAID. We posit that HCPs contemporaneously undergo the endogenous process of reconciliation and the exogenous process of intentional contemplation in determining their non-participation threshold. The factors influencing non-participation are fluid and may shift or evolve as HCPs’ personal and professional experiences change, and as such, HCPs’ non-participation threshold may also change. Alternative mechanisms to support HCPs’ and patients’ mutual expectations in the social contract of care are required if HCPs continue as non-participators in formal MAID processes. However, the shifting or evolving factors may also culminate in HCPs’ participation in formal MAID processes, including MAID provision. The social contract expectations between the requesting patient and the participating HCP are met in this instance.

### Implications for Practice

There may be an opportunity to mitigate some of the exogenous factors that influenced HCPs’ non-participation in formal MAID processes. The considerations below are not intended to compel nor convince HCPs to participate; however, they may support those HCPs who are considering formal participation but are reluctant or unable to do so. Specifically, we suggest clarifying the regional model of care, practice-focused MAID education, policy clarification, time, and practice enhancements.

#### Clarifying the regional model of care

Each province and territory is responsible for delivering health care services, and, not surprisingly, each has developed a distinct regional MAID model of care (Health Canada, 2020; Silvius et al., 2019). Some MAID models have a central access point and dedicated teams and resources, where others have incorporated MAID into the existing workload of the HCP. HCPs, in our research, expressed uncertainty about how MAID “fit” in their practice. Clarifying and communicating the operational aspects of the regional MAID model of care is urgently required so that HCPs can accurately contemplate their obligations, ideals, and participation consequences, ensuring their perspectives are constructed on the regional practice model.

#### Practice-focused MAID education and policy clarification

Practice-focused education and policy clarification may also support HCPs who are intentionally contemplating formal participation but are reluctant or unable to do so. This includes policy and process clarification (i.e., how to obtain the MAID provision medications, how to complete death certificates, and other related administrative practices), education that moves beyond the legislative framework of MAID, and support for HCPs who do wish to engage in such education. MAID is a complex process (Brooks, 2019) with a significant “learning curve” (McKee & Sellick, 2018, p. e89). This complexity and learning curve of MAID, in addition to our findings related to competency and lack of knowledge, signals that enhanced MAID education is required. Knowledge of the medical-legal and technical aspects of participation in MAID processes, communication skills, information on religion and MAID, explicit information on roles and responsibilities, and an opportunity to clarify personal feelings regarding MAID were desired by nursing and medical students (Bator et al., 2017; McMechan et al., 2019). As identified in this research, this same level of detailed and specific practice-focused information would support all HCPs as they intentionally contemplate their degree of participation in formal MAID processes.

#### Time

HCPs’ non-participation in formal MAID processes was influenced by competing priorities in a timed clinic visit and their belief that participation in formal MAID processes required time beyond what they had available. Adequate time is a crucial foundational element in all patient–HCP relationships (Braddock & Snyder, 2005), and relationships are critical in MAID processes (Brooks, 2019). To ensure the promotion of ongoing excellent care, HCPs and patients need time for safe and satisfying clinical encounters. The need for adequate time to discuss EOL care with patients and families and, for those who desire, to participate in formal MAID processes is acute as MAID deaths are increasing in Canada (Health Canada, 2020) and the Canadian population continues to increase and age (Statistics Canada, 2019). System-wide action is required to ensure that HCPs (regardless of MAID participation) have adequate time to provide relational, holistic patient care and that practices in rural and remote areas have sufficient HCPs to meet the care health care needs of the population.

#### Practice enhancements

Some non-participation considerations may be mitigated through practice enhancements such as fair remuneration, clear professional guidance, systems that respond to safety and risk concerns, and removal of practice barriers. Khoshnood et al. (2018) identified that MAID assessors and providers were concerned about remuneration, echoing our research. Given the practice, time, and relational investments of participation in formal MAID processes, reviewing remuneration policies for physicians and NPs is clearly warranted.

HCPs, in our research, considered the professional and legal risk of participation. This risk may stem from the often-polarized discourse surrounding the interpretation and application of the legislation. For example, HCPs can inform patients about MAID as an EOL care option but cannot say anything that could be construed as counseling someone toward an assisted death (Pesut et al., 2019). Clear professional guidance regarding the legal and professional bounds of MAID may assist HCPs in assessing the risk of participation. Professional associations and employers must respond to concerns regarding the physical, emotional, and mental safety of the HCPs and their families and provide both support and action such that risks are mitigated, and healthy workplaces are supported. Our data were collected approximately 3 years after MAID legalizations, and these considerations regarding risk may shift as the Canadian experience with MAID continues.

Finally, NPs encounter many systemic barriers to their practices (Hain & Fleck, 2014), and NPs in our research identified practice limits or barriers that influenced their non-participation in formal MAID processes. A concerted review to mitigate NPs practice barriers is crucial so NPs may work to their full scope of practice in a respectful work environment. This would include (a) reviewing employer job descriptions to support those who may wish to participate in MAID, (b) ensuring remunerations structures support NPs formal participation in MAID processes, (c) ensuring NPs can roster patients in their practices to develop sustained relationships, (d) allowing NPs to admit patients to hospitals, and (e) actively counteracting outdated perceptions of what a full-scope NP practice entails.

Additional future research could explore if and how the factors and decision-making considerations vary by HCP sub-group, practice location, region, or over time. An inquiry into Canadians’ perspectives from diverse cultural backgrounds and faiths regarding MAID would contribute to improved working relationships with diverse patient populations. Finally, it is important to ascertain the efficacy of the proposed mitigations in positively supporting the HCPs who might have considered formal participation but were reluctant or unable to do so.

### Limitations

We acknowledge that within our epistemology, additional data or variations within the data exist. Our qualitative interpretations are specific to the time (data collected approximately 3 years after MAID legalization in Canada), place, and participants of this research; thus, we have provided detailed accounts of the participants to support transferability. Despite the use of vignettes in the data collection, the majority of the participants’ responses were hypothetical as only 27% of them had encountered an actual patient request for MAID. The research regarding HCPs’ participation in MAID processes is emerging; thus, we utilized research from international jurisdictions to position our findings, which may differ from Canadian health care delivery approaches, culture, and laws.

## Conclusion

Accounting for the reasoning of HCPs within their personal, patient, practice, and community contexts is vital to understand non-participation in ethically complex care. The factors and decision-making considerations influencing HCPs’ non-participation in formal MAID processes are extensive. Referral pathways that align with HCPs’ moral space and are sufficiently known to all patients, family members, and health care team members will support the social contract between HCPs and patients at the EOL. Clarifying the regional MAID model of care, practice-focused education, policy clarification, time, and removal of practice barriers may support those HCPs who may consider formal participation in MAID processes but are reluctant or unable to do so. Supporting these HCPs may, in turn, foster sustainability in MAID programs and support the social contract of care by facilitating patients’ access to MAID.

## Supplemental Material

sj-pdf-1-qhr-10.1177_10497323211027130 – Supplemental material for “I Am Okay With It, But I Am Not Going to Do It”: The Exogenous Factors Influencing Non-Participation in Medical Assistance in Dying Click here for additional data file. Supplemental material, sj-pdf-1-qhr-10.1177_10497323211027130 for “I Am Okay With It, But I Am Not Going to Do It”: The Exogenous Factors Influencing Non-Participation in Medical Assistance in Dying by Janine Brown, Donna Goodridge, Lilian Thorpe and Alexander Crizzle in Qualitative Health Research

sj-pdf-2-qhr-10.1177_10497323211027130 – Supplemental material for “I Am Okay With It, But I Am Not Going to Do It”: The Exogenous Factors Influencing Non-Participation in Medical Assistance in Dying Click here for additional data file. Supplemental material, sj-pdf-2-qhr-10.1177_10497323211027130 for “I Am Okay With It, But I Am Not Going to Do It”: The Exogenous Factors Influencing Non-Participation in Medical Assistance in Dying by Janine Brown, Donna Goodridge, Lilian Thorpe and Alexander Crizzle in Qualitative Health Research
