# Supplementary material for: “I Am Okay With It, But I Am Not Going to Do It”: The Exogenous Factors Influencing Non-Participation in Medical Assistance in Dying
Source: Qual Health Res. 2021 Jul 8;31(12):2274–89. doi: 10.1177/10497323211027130 (PMC8564235; doi:10.1177/10497323211027130)
Supplement: sj-pdf-1-qhr-10.1177_10497323211027130 – Supplemental material for “I Am Okay With It, But I Am Not Going to Do It”: The Exogenous Factors Influencing Non-Participation in Medical Assistance in Dying [file sj-pdf-1-qhr-10.1177_10497323211027130.pdf]

Supplemental File 1: Semi-structured Interview Guide and Vignettes

**Introductory Questions**	**Follow-up Questions:**
Tell me about your current practice:	o How long have you been in practice? What is the age range of patients typically cared for? What proportion of patients have life-limited illnesses? What type of life-limiting illness do you see?<br>o How many of your patients have died over the previous month? In the last year?<br>Tell me about your understanding of the MAID legislation.
In your current practice, do you routinely participate in end-of-life care discussions?	o If yes, are these discussions patient or practitioner initiated? How have end-of-life care discussions (with your patients, your colleagues, your families) changed since MAID legalization?<br>o What is it like to work in your practice setting since MAID legalization?
Which one of the statements best reflects your feelings regarding participation in MAID related care:<br>A) "I don't know what I would do if approached."<br>B) "I don't think I would participate."<br>C) "I might be interested in participating."<br>D) "I would not participate."	<u>If answered A, B or C</u><br>o Tell me about your hesitation.<br>o What is making you unsure?<br>o How could colleagues, patients, regulators, and/or health system leadership support you in working through your hesitations or uncertainty?<br><u>If answered D,</u><br>o Tell me about your thoughts that brought you to your choice.<br>o Are there any circumstances when you would consider participation? If so, what would they be?
**Vignette #1**	
Vignette Part 1 Introduction and Background:<br><br>You are the care provider for a 67-year old patient who was diagnosed four years ago with stage IV metastatic breast cancer. At that time, the patient underwent a bilateral mastectomy and follow-up oncology care. Recently, the patient presented with headaches, drowsiness, and vision changes. An MRI was arranged and discovered a significant malignant frontal lobe brain lesion. Specialists believed it might be partially resected and reduced further with subsequent treatment. After consulting with her adult child and spouse, the patient requested palliative care. This was arranged.<br><br>Part 1: On a follow-up appointment, the patient states she is very pleased with palliative care and her symptom control. However, she notes that she is rapidly losing her vision. She asks you for information on MAID, including eligibility and how to access care.	o Do you provide information regarding MAID to your patient? Tell me about your choice.<br>o If no, do you consider referring the patient to another practitioner?<br>o What factors are particularly difficult to consider?

Part 2: This patient now presents for a follow-up appointment and arrives with MAID information. She asks you for a formal assessment to determine if she would qualify for MAID.	o Do you provide an assessment? Tell me about your choice.<br>o What factors are particularly difficult to consider?
Part 3: This patient has since been found eligible for MAID through the assessment process. As one of her care providers, she asks you to be present on the day of her chosen death to provide emotional support to her and her family.	o Do you agree to be there? Tell me about your choice.<br>o What factors are particularly difficult to consider?
Part 4: The patient asks you to be her MAID provider and administer the medications.	o Do you agree to be a provider? Tell me about your choice.<br>o What factors are particularly difficult to consider?
**Vignette #2***	
Vignettes Part 1 Introduction and Background:<br><br>You are at a professional development conference and part of a physician panel of experts discussing the Canadian Medical Association Code of Ethics and Professionalism in emerging practice areas.<br><br>Part 1: Your group is discussing the physician's responsibility to "consider first the well-being of the patient" when a patient requests MAiD.	o How do you explain the Code of Ethics in relation to MAiD?
Part 2: During the question and answer section of the panel discussion, a conference participant asks, "how do physicians practice professionally when their personal beliefs do not align with the care a patient requests?"	o How do you respond?<br>o What factors are particularly difficult to consider?
Part 3: During the conference nutrition break, the discussion continues at your table. One colleague states, "physicians have a duty to respect the patient's choice." Another colleague states, "physicians can choose what care they provide." They turn to you and ask you your thoughts.	o How do you respond to your colleagues?<br>o How easy or difficult is it for you to participate in this conversation with your colleagues?<br>o How does conscientious objection fit into these conversations?
**Vignette #3**	o
The health authority, in partnership with your professional regulator, is considering a continuing education opportunity to support practitioners concerning MAiD care provision.<br><br>Part 1: How likely is it you participate in this training? Tell me about your choice.	o What is holding you back from participating?
Part 2: How do you view this training and education in relation to your current practice?	o What factors are particularly difficult to consider?
**Concluduing Question**	**Follow-up Questions:**
Is there anything else you would like to tell me that I have not thought to ask?	o

* Amended to "Promote Health and Well-being" in alignment with the Code of Ethics for Registered Nurses for NP participants
