# Supplementary material for: “I Am Okay With It, But I Am Not Going to Do It”: The Exogenous Factors Influencing Non-Participation in Medical Assistance in Dying
Source: Qual Health Res. 2021 Jul 8;31(12):2274–89. doi: 10.1177/10497323211027130 (PMC8564235; doi:10.1177/10497323211027130)
Supplement: sj-pdf-2-qhr-10.1177_10497323211027130 – Supplemental material for “I Am Okay With It, But I Am Not Going to Do It”: The Exogenous Factors Influencing Non-Participation in Medical Assistance in Dying [file sj-pdf-2-qhr-10.1177_10497323211027130.pdf]

Supplemental File 2: Contextual Data of Participants (N=35)

Contextual Data	Would Not Participate Beyond a Referral n = 14 (40%*)	Participants Who Would Provide More than a Referral, but not Formally Administer MAID n = 21 (60%*)
Nurse Practitioner	6 (43%)	12 (57%)
Physician	8 (57%)	9 (43%)
Female	9 (64%)	14 (67%)
Male	5 (36%)	7 (33%)
Single/Never Married	1 (7%)	-
Married/Domestic Partnership	13 (93%)	17 (81%)
Divorced	-	4 (19%)
Age (years):		
• 25-34	3 (21%)	2 (10%)
• 35-44	4 (29%)	5 (24%)
• 45-54	2 (14%)	8 (38%)
• 55 and older	5 (36%)	6 (29%)
Years in Practice:		
• 1-9	6 (43%)	6 (29%)
• 10-19	3 (21%)	8 (38%)
• 20-29	4 (29%)	4 (19%)
• 30-39	1 (7%)	3 (14%)
Significance of faith, religions,		
spirituality:	7 (50%)	-
• Extremely Significant	3 (21%)	5 (24%)
• Very Significant	2 (14%)	2 (10%)
• Significant	2 (14%)	11 (52%)
• Neutral	-	3 (14%)
• Not Significant		
Belief system:		
• Protestant	4 (29%)	3 (14%)
• Non-denominational Christianity	3 (21%)	3 (14%)
• Agnostic/Atheist	-	4 (19%)
• Islam	1 (7%)	-
• Roman Catholic	5 (36%)	8 (38%)
• Did not disclose/Other	1 (7%)	3 (14%)
Location of Practice:**		
• Large Population Centre	5 (36%)	11 (52%)
• Medium Population Centre	1 (7%)	2 (10%)
	3 (21%)	6 (29%)

• Small Population Centre<br>• Rural area	5 (36%)	2 (10%)
Primary Work Area:<br>• Family Medicine/Primary Care<br>• Specialty Practice Areas***	 8 (57%)<br>6 (43%)	 13 (62%)<br>8 (38%)
Patients with Life-Limiting Illness (%):<br>• 0-19%<br>• 20-39%<br>• 40% or more	 8 (57%)<br>4 (29%)<br>2 (14%)	 15 (71%)<br>4 (19%)<br>2 (10%)
Responses in the interview were informed by:<br>• An actual patient request<br>• A hypothetical request	 4 (29%)<br>10 (71%)	 5 (24%)<br>16 (76%)

* May equate 100% due to rounding.

** The Statistics Canada definitions were provided to the participants to support selection.

*** Including, but not limited to, emergency medicine, internal medicine, anesthesiology, surgery, critical care, psychiatry, and physical and rehabilitation medicine.
